# Correlation between cerebral hemodynamic functional near-infrared spectroscopy and positron emission tomography for assessing mild cognitive impairment and Alzheimer’s disease: An exploratory study

**DOI:** 10.1371/journal.pone.0285013

**Published:** 2023-08-10

**Authors:** Jin A. Yoon, In Joo Kong, Ingyu Choi, Jihyun Cha, Ji Yeong Baek, JongKwan Choi, Yong Beom Shin, Myung Jun Shin, Young-Min Lee

**Affiliations:** 1 Department of Rehabilitation Medicine, Pusan National University School of Medicine and Biomedical Research Institute, Pusan National University Hospital, Busan, Republic of Korea; 2 OBELAB Inc., Seoul, Republic of Korea; 3 Department of Psychiatry, Pusan National University School of Medicine and Biomedical Research Institute, Pusan National University Hospital, Busan, Republic of Korea; Nathan S Kline Institute, UNITED STATES

## Abstract

This study was performed to investigate the usefulness of functional near-infrared spectroscopy (fNIRS) by conducting a comparative analysis of hemodynamic activation detected by fNIRS and positron emission tomography (PET) and magnetic resonance imaging (MRI) in patients with mild cognitive impairment (MCI) and Alzheimer’s disease (AD). Participants were divided into four groups: the subjective memory impairment (SMI), amnestic MCI (aMCI), non-amnestic MCI (naMCI), and AD groups. We recorded the hemodynamic response during the semantic verbal fluency task (SVFT) using a commercial wireless continuous-wave NIRS system. The correlation between the parameters of the neuroimaging assessments among the groups was analyzed. Region of interest-based comparisons showed that the four groups had significantly different hemodynamic responses during SVFT in the bilateral dorsolateral prefrontal cortex (DLPFC). The linear mixed effect model result indicates that the mean *ΔHbO*_*2*_ from the bilateral DLPFC regions showed a significant positive correlation to the overall FDG-PET after controlling for age and group differences in the fNIRS signals. Amyloid PET signals tended to better differentiate the AD group from other groups, and fNIRS signals tended to better differentiate the SMI group from other groups. In addition, a comparison between the group pairs revealed a mirrored pattern between the hippocampal volume and hemodynamic response in the DLPFC. The hemodynamic response detected by fNIRS showed a significant correlation with metabolic and anatomical changes associated with disease progression. Therefore, fNIRS may be considered as a screening tool to predict the hemodynamic and metabolic statuses of the brain in patients with MCI and AD.

## Introduction

Alzheimer’s dementia is the most common type of neurodegenerative disease, accounting for approximately 70% of all cases of dementia [1]. It is estimated that 15–20% of adults aged 65 years or more have mild cognitive impairment (MCI) [2], which progresses to Alzheimer’s dementia in approximately 30% of the cases. Currently, it is reported that initiating drug therapy at the stage of MCI is the most effective for slowing its progression to Alzheimer’s dementia [3]. The two-hit hypothesis, a hypothesis based on the combination of the vascular and amyloid cascade, postulates that amyloid-beta (Aβ) deposition, which occurs after vascular dysfunction, causes neurodegeneration and cognitive decline during the progression from MCI to Alzheimer’s dementia [4]. Accordingly, cerebral hypoperfusion is an important biomarker for determining the presence of the disease, as well as the risk for progression [3,5]. Positron emission tomography (PET) and single-photon emission computed tomography imaging are useful for assessing the metabolic activity accompanying cerebral blood perfusion and predicting the progression of MCI to Alzheimer’s disease (AD) with high sensitivity and specificity [6–8]. However, imaging takes a long time and is expensive. Thus, it is often the case that testing is not performed at the right time, and patients are not diagnosed and treated early. Multi-channel functional near-infrared spectroscopy (fNIRS) is a promising alternative for the early diagnosis of AD and MCI. Several studies have examined hemodynamic responses during MCI and AD using fNIRS and showed decreased levels of activation in specific brain regions of patients with AD relative to the control group [9–12]. In addition, fNIRS has also been used to evaluate hemodynamic impairments in AD during the resting state, which are unrelated to functional activity [13,14].

Therefore, by confirming the pattern of hemodynamic responses in AD and MCI, useful biomarkers assessable by fNIRS for the early diagnosis of MCI may be discovered. PET assesses changes in metabolic activity accompanied by changes in cerebral blood flow [15]. Establishing the relationships between metabolic activity, structural changes, and amyloid plaque load detected with PET and magnetic resonance imaging (MRI) and hemodynamic responses detected with fNIRS would help in evaluating the reliability of fNIRS. However, no studies have been conducted to investigate these relationships. The present study aimed to investigate the usefulness of fNIRS by conducting a comparative analysis of hemodynamic activation detected by fNIRS and PET in patients with MCI and AD.

## Materials and methods

### Study participants

The study participants were patients aged 60 years or more who visited the psychiatry clinic of the study hospital due to cognitive impairment between June 2015 and July 2019. A final diagnosis was confirmed by a psychiatrist using a multidisciplinary approach involving medical examination, neuropsychological and neuroimaging assessments, and neurocognitive tests such as the Seoul Neuropsychological Screening Battery (SNSB) 2nd edition, brain MRI, and F-18 (flutemetamol) amyloid PET. Based on the results of the evaluation, the participants were allocated to the subjective memory impairment (SMI), amnestic MCI (aMCI), non-amnestic MCI (naMCI), and AD groups. Patients with AD met the National Institute of Neurological and Communication Disorders and Stroke and the Alzheimer’s Disease and Related Disorders Association criteria for probable AD [16]. Patients with MCI met the following Petersen’s criteria [17]: (a) subjective memory complaints by patients or caregivers, (b) normal activities of daily living (ADL) based on clinical findings and the ADL scale (S-IADL) [18], (c) objective memory impairment based on the word list delayed recall test (Seoul Verbal Learning Test, delayed recall) as evidenced by a Z-score of 1.5 below the mean of the age-, sex- and education-matched normal individuals, and (d) the absence of dementia. Patients with (a) above-moderate dementia severity (Clinical Dementia Rating ≥ 2); (b) an axis I diagnosis of delirium, schizophrenia, bipolar disorder, and major depressive disorder; (c) clinically active cerebrovascular disease (e.g., stroke within 6 months, multiple lacunae, and severe white matter hyperintensities with Fazekas scale score = 3) or other conditions causally related to cognitive impairment (e.g., severe organ failure, metabolic or hematologic disorders and clinically significant abnormal laboratory findings); and (d) other neurodegenerative disorders (dementia with Lewy bodies, frontotemporal dementia, and Parkinson’s disease) were excluded. This study was approved by the Institutional Review Board of our hospital (IRB No. 2204-001-113). All methods were performed in accordance with the relevant guidelines and regulations. The patients were enrolled in the study after they had provided written informed consent.

### fNIR data acquisition and processing

A total of 107 participants were divided into four groups: SMI (n = 24), aMCI (n = 30), naMCI (n = 29), and AD (n = 24). We recorded the hemodynamic response during the protocol using a commercial wireless continuous-wave NIRS system (OBELAB Inc., Seoul, Republic of Korea) [19,20].

**Fig 1:**
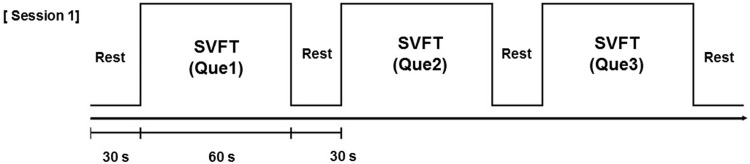
Cognitive task protocol used for the NIRSIT system. SVFT, semantic verbal fluency task.

Participants wore the NIRS system, the neuroimaging device for fNIRS, and performed a verbal fluency task. The task was administered over three sessions, with a 30-s break between sessions (Fig 1). Semantic verbal fluency task (SVFT) [21] involves generating as many words as possible within a certain time frame and providing hints about the semantic category of the words generated. The amount of information related to categorization and the number of words that can be retrieved from memory within 1 min are determined. In this study, the task involved generating as many words related to the keywords as possible.

**Fig 2:**
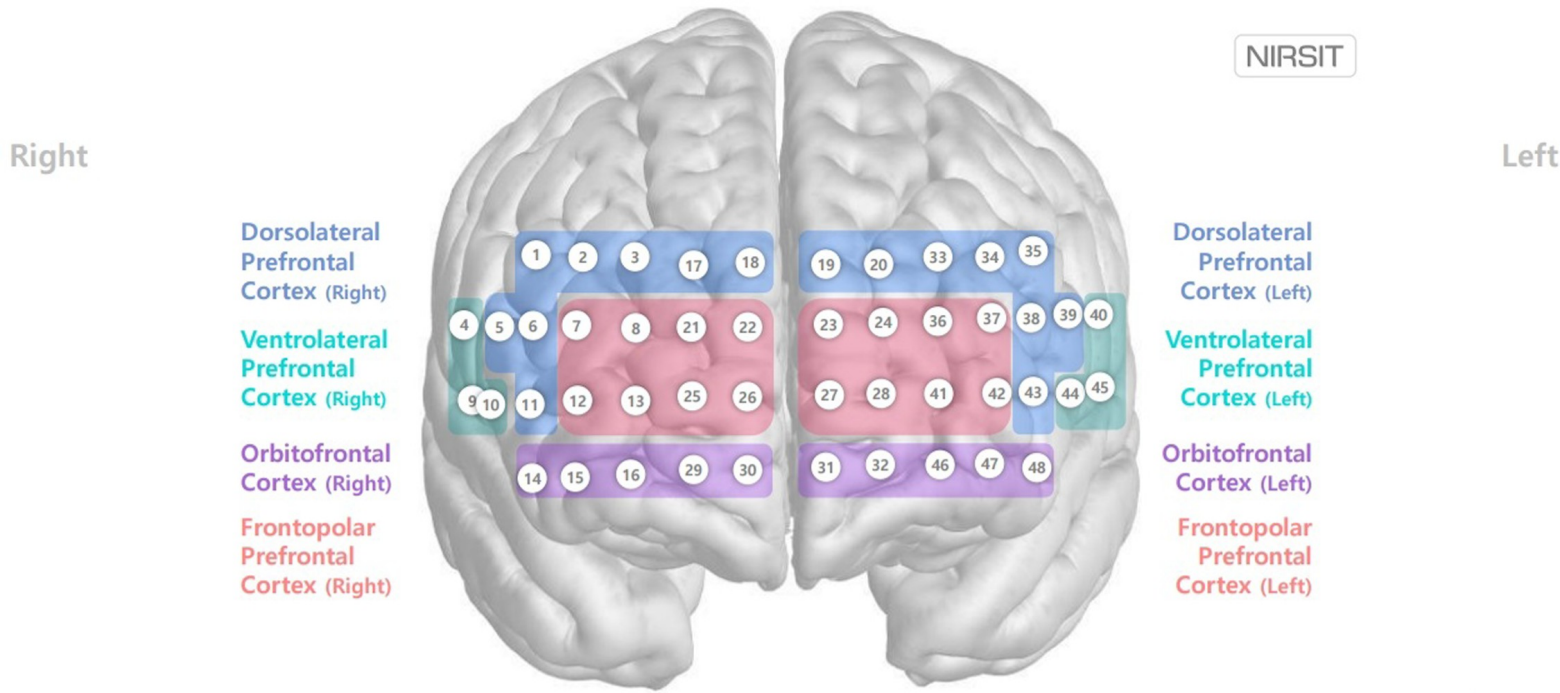
Schematic channel position on normalized brain atlas.

Hemodynamic response of the prefrontal cortex was recorded using a high-density NIRS device which was composed of 24 sources (laser diodes) emitting two wavelengths (780/850 nm) and 32 photo-detectors, at a sampling rate of 8.138 Hz [22]. The total number of channels was 48, and the detected light signals for each wavelength were filtered by a band-pass filter (0.005–0.1 Hz) to minimize environmental noise-related light and physiological noise due to body movement (Fig 2). The poor-quality channels (signal-to-noise ratio<30 dB) were rejected before extraction of the hemodynamics data to prevent misinterpretation. The relative hemodynamic changes of each channel during each trial of the tasks were assessed separately using the modified Beer–Lambert law [23]. The results of multiple trials were block-averaged individually before being grand-averaged for each group. As the BOLD-oxygenated hemoglobin (oxy-Hb/HbO_2_) correlation showed higher correlation than BOLD-deoxy-Hb correlation in previous study [24], the oxy-Hb/HbO_2_ values (ΔHbO_2_) during the task period were measured as representing the activation of the prefrontal cortex. To correct the motion artifacts due to the differential pathlength factor of the participants, correlation-based signal improvement (CBSI) correction was performed before analysis after measurement [25].

### MRI data acquisition and image analysis

Each participant underwent a structural T1-weighted MRI and a diffusion tensor imaging scan at the clinical evaluation. All images were acquired at Pusan National University Hospital Imaging Center on a Siemens 3-T Trio TIM scanner (Erlangen, Germany).

For each participant’s cortical thickness or volume estimation, a 3-D magnetization-prepared rapid gradient echo sequence was acquired using the following parameters: repetition time = 1800 ms, echo time = 2.07 ms, flip angle = 12˚, acquisition matrix = 256 × 256, field of view = 250 × 250 mm^2^, slice thickness = 1 mm, and the total number of slices = 256.

All image acquisitions had the same slice orientation parallel to the anterior commissure and posterior commissure line. Motion was restricted with expandable foam cushions. Scans with movement or any other image (reconstruction) artefacts were excluded.

The FreeSurfer version 5.1 software package (http://surfer.nmr.mgh.harvard.edu/) was used to analyze the cortical thickness or volume on 3-D T1-weighted images [26]. Each brain region was identified using the Desikan–Killiany Atlas [27]. We visually examined all images to ensure segmentation accuracy.

### Florbetaben PET acquisition and imaging analysis

All participants underwent amyloid PET scans with a Biograph 40 PET/CT scanner (Siemens, Knoxville, TN, USA). Amyloid PET data were acquired between 90 and 110 mins after the injection of 185 MBq of ^18^F-florbetaben. Amyloid PET images were reconstructed using the iterative ordered-subset expectation maximization algorithm [28].

Quantitative analysis was conducted by normalizing the MRI images to a T1-weighted MRI template and co-registering PET images with the MRI images in all cases. Each brain region was identified using the Automated Anatomical Labeling atlas [29]. We calculated the level of Aβ deposition in each brain region and expressed it as standard uptake value ratios (SUVRs), by using the whole cerebellum as a reference region. Amyloid status (Aβ positive or negative) was determined using a previously described method based on a visual assessment by a nuclear physician blinded to clinical data [30].

### ^18^F-FDG PET acquisition and imaging analysis

All participants underwent PET scans using a Biograph 40 PET/CT scanner (Siemens, Knoxville, TN, USA). PET data were acquired 60 mins after the injection of 4.8 MBq/kg of ^18^F-FDG. PET images were reconstructed using the iterative ordered-subset expectation maximization algorithm. Quantitative analysis was conducted by normalizing the MRI images to a T1-weighted MRI template and co-registering PET images with the MRI images in all cases. We calculated the brain glucose metabolism of each brain region and expressed it as SUVRs, using the global brain area as a reference.

### Statistical analysis

#### Region of interest

Before performing any statistical tests, the mean Δ*HbO*_2_ of each channel during SVFT was averaged to form eight Brodmann regions to yield more stable signals from each participant and to reduce the number of comparisons in subsequent tests. The resulting eight regions of interest (ROIs) include the right and left dorsolateral prefrontal cortex (DLPFC), right and left frontopolar cortex, right and left orbitofrontal cortex (OFC), and right and left ventrolateral prefrontal cortex (VLPFC). Since the majority of the channels in bilateral VLPFC were rejected during the preprocessing step, formal statistical tests were performed on regions other than bilateral VLPFC.

Due to the exploratory nature of the study, we started with all features within the dataset, including 6 fNIRS Brodmann regions, 8 aPET measures, 8 fluorodeoxyglucose (FDG)-PET measures, 88 volume measures, and 68 thickness measures, totaling 180. From this dataset, two sets of fNIRS regions were selected as fNIRS ROIs: (1) right and left OFC, where participants (collapsing across groups) showed significant activation during SVFT, and (2) right and left DLPFC, where the main effect of group was significant, demonstrating a greater activation in the SMI group than other groups. The analysis of variance (ANOVA) results are elaborated in the results section.

#### Feature selection

Two formal tests were used to select the features to be submitted for further analyses. First, as a formal test to investigate the relationship between the mean Δ*HbO*_2_ from the fNIRS and other neurological measures, linear mixed effect (LME) models predicting the mean Δ*HbO*_2_ with each measure were fitted, including subjects’ age as a covariate and the random intercept of the group factor to account for differences in the fNIRS signal across the group that can potentially overshadow the relationship between the two measures. Thus, the LME model for the formal test is shown below.

*fNIRS (mean ΔHbO*_*2*_*) ∼ B*_*0*_*+B*_1_* Neurological Measurement + +B_2_*Age + (1|Group). Second, multivariate analysis of covariance (MANCOVA) tests were performed to see if the group factor (diagnosis) had any differential effect on fNIRS signals and neurological measures, implying that fNIRS can potentially provide additional information above and beyond what conventional neurological measures could provide. **MANCOVA was selected to reduce the number of tests required and to scope in detail in a top-down method, and a method of selecting and reporting areas and indicators that showed significant effects in this model was selected.** In reporting the test results, when both the right and left parts of the same fNIRS ROI were statistically significant, the mean of the two ROIs was averaged, and the result from the bilateral ROIs was reported. Otherwise, original results from either right or left ROI were illustrated. The threshold for all statistical significance was set at *p* < .05.

## Results

Table 1 shows the number of subjects in each group who had data from each imaging study. The sample size for each test (either LME or MANOVA) varied because the number of subjects who had fNIRS and other measurements differed across the pairs of methods to be compared. Additionally, the outliers beyond the 1.5 interquartile range within each group for each comparison method were also removed per analysis.

**Table 1:** The number of subjects in the full data set who had measurements from each domain.

Group	fNIRS	aPET	FDG-PET	Deep Gray Volume	Gray Volume	Cortical Thickness
SMI	24	24	21	24	19	24
naMCI	29	23	18	27	25	27
aMCI	30	27	25	28	24	28
AD	24	23	22	24	14	24
Total	107	97	86	103	82	103

SMI: Subjective memory impairment, naMCI: Non-amnestic mild cognitive impairment, aMCI: Amnestic mild cognitive impairment, AD: Alzheimer’s disease, fNIRS: Functional near-infrared spectroscopy, aPET: Positron emission tomography, FDG-PET: Fluorodeoxyglucose-positron emission tomography.

There were no significant differences in age, sex ratio, or education level between the groups (all *p* values < .05) (Table 2). The results of the initial neuropsychological test were significantly different between the groups (Table 3).

**Table 2:** Clinical characteristics of patients.

	SMI (n = 24)	naMCI (n = 29)	aMCI (n = 30)	AD (n = 24)	*p*-value
Age (yrs)	73.4 ± 4.8	73.6 ± 5.3	74.0 ± 4.7	74.8 ± 6.0	0.79
Sex					0.26
Male	6	6	13	8	
Female	18	23	17	16	
Education (yrs)	7.6 ± 4.3	7.4 ± 4.3	8.9 ± 5.2	7.6 ± 4.3	0.60

SMI: Subjective memory impairment, naMCI: Non-amnestic mild cognitive impairment, aMCI: Amnestic mild cognitive impairment, AD: Alzheimer’s disease.

**Table 3:** Comparison of neuropsychological test results of the groups.

Neuropsychological tests	SMI (n = 24)	naMCI (n = 29)	aMCI (n = 30)	AD (n = 24)	*p*-value
Language function					
K-BNT	11.3 ± 2.8	9.7 ± 2.9	9.7 ± 2.8	7.8 ± 3.0	0.001
Memory					
RCFT: delayed recall	11.7 ± 7.1	5.6 ± 4.8	6.0 ± 5.9	1.1 ± 1.9	0.000
Frontal/executive functions					
TMT: part B	84.6 ± 76.1	167.5 ± 96.7	148.0 ± 103.8	213.7 ± 98.0	0.00
STROOP: color reading	70.2 ± 21.1	37.1 ± 20.9	47.2 ± 28.7	26.9 ± 21.3	0.00

K-BNT: Korean-Boston Naming Test, RCFT: Rey Complex Figure Task, TMT: Trail Making Test, SMI: Subjective memory impairment, naMCI: Non-amnestic mild cognitive impairment, aMCI: Amnestic mild cognitive impairment, AD: Alzheimer’s disease.

**Fig 3:**
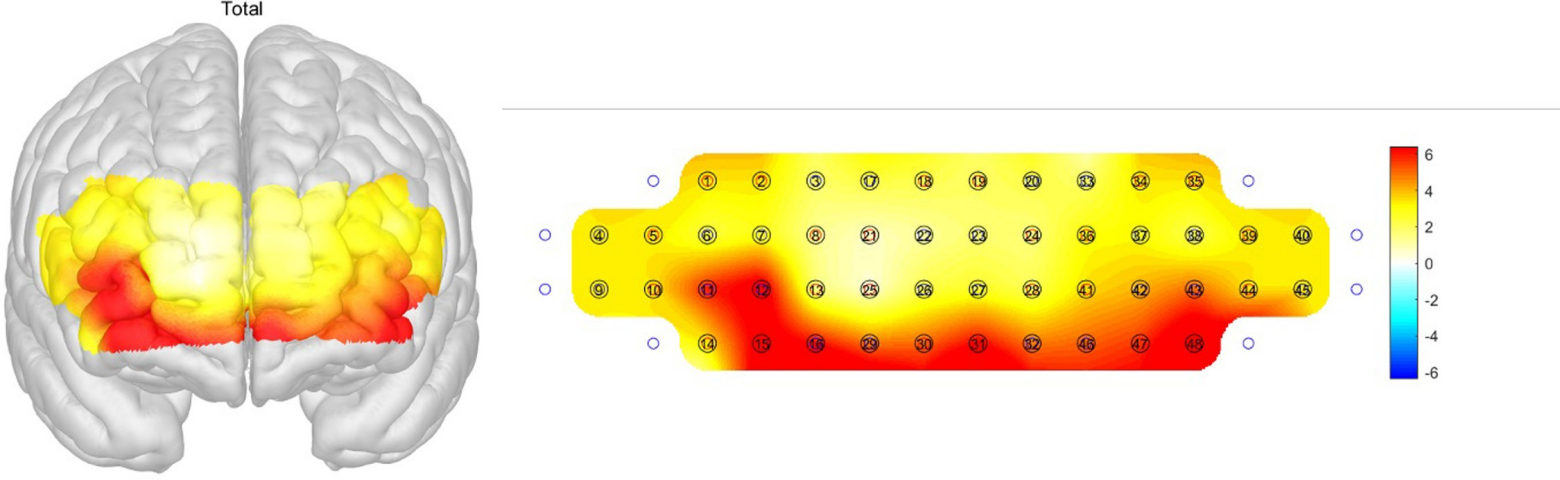
fNIRS activation (t-values) during the SVFT task, where the warmer color indicates a greater activation. The ventral part of the PFC involving the bilateral orbitofrontal cortex showed significant activation. fNIRS: Functional near-infrared spectroscopy, SVFT, semantic verbal fluency task, PFC: Prefrontal cortex.

**Fig 4:**
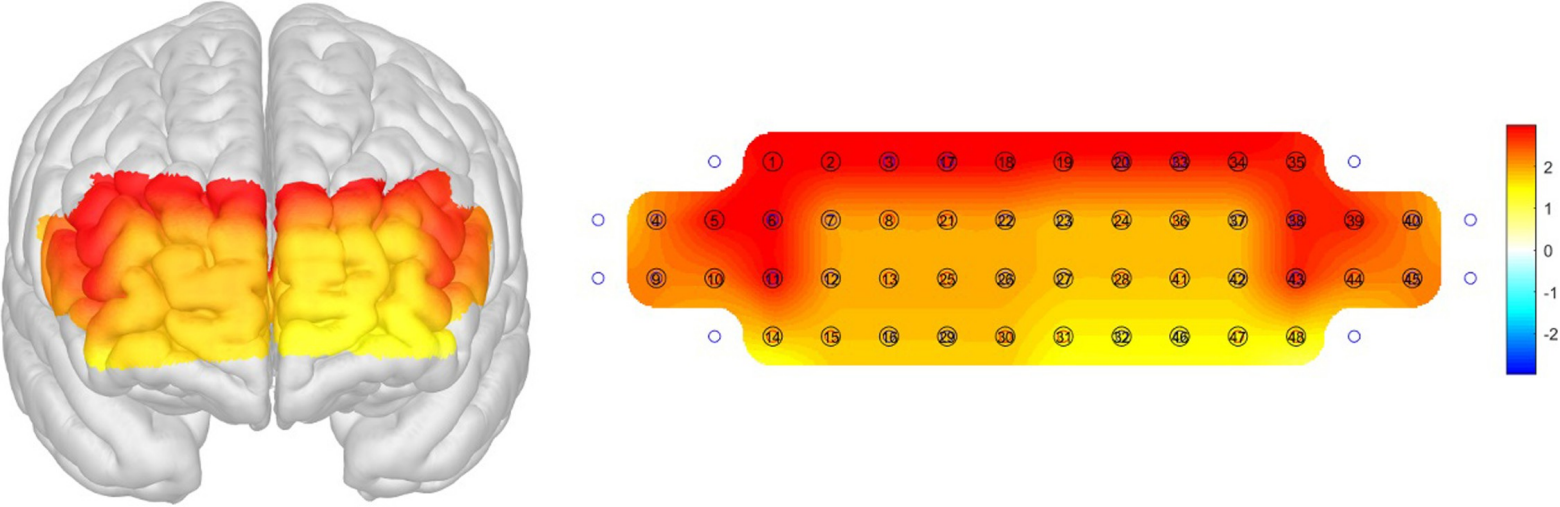
fNIRS group effect map (F-values) showing differential group activation during SVFT, where the warmer color indicates greater group difference. The dorsal part of the PFC involving the bilateral dorsolateral cortex shows a significant group effect. fNIRS: Functional near-infrared spectroscopy, SVFT, semantic verbal fluency task, PFC: Prefrontal cortex.

**Fig 5:**
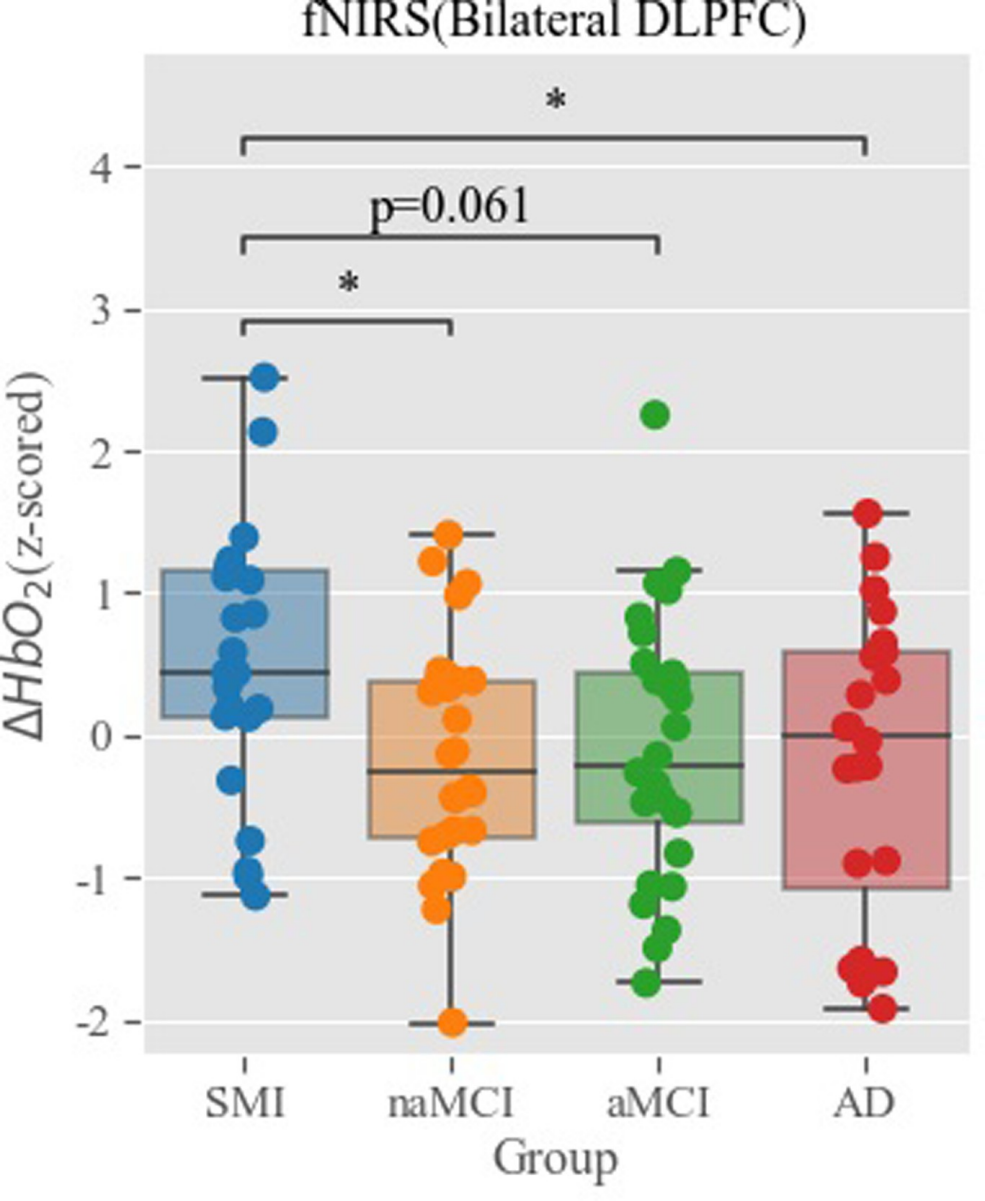
Comparison of the mean Δ*HbO*_2_ during SVFT between groups. SVFT, semantic verbal fluency task.

Bilateral OFC ROIs showed significant activation during the SVFT test when the subjects were collapsed across groups demonstrating that the orbitofrontal cortex is the functional ROI that showed a task-related activation [*t*(105) = 8.77, *p*<0.001) (Fig 3). Furthermore, one-way between-subject ANCOVA was conducted to examine the effect of the diagnosed group on the mean *ΔHbO*_*2*_ of each region after controlling for subject age. The main effect of the group was significant in the left DLPFC [*F*(3, 101) = 2.79, *p* = 045], right DLPFC, [*F*(3, 99) = 2.85, *p* = 041], and bilateral DLPFC as a whole [*F*(3, 98) = 3.01, *p* = 034] (Fig 4). Dunnett’s post-hoc test on groups revealed that the SMI group showed greater bilateral DLPFC activation during SVFT than the naMCI (*p* = .032), AD (*p* = .027), and aMCI groups (marginally greater; *p* = .061) (Fig 5).

### fNIRS and FDG-PET

The LME model result indicates that the mean *ΔHbO*_*2*_ from the bilateral DLPFC regions showed a significant positive correlation to the overall FDG-PET controlling for age and group difference in the fNIRS signals. Table 4 summarises the model output. This relationship was consistent (in terms of statistical significance and the size of association) across regions where the fNIRS and FDG-PET signals were collected. Importantly, one-way ANCOVA testing the group diagnostic effect on the global FDG-PET, with age as a covariate, revealed that the FDG-PET signal did not differ across groups, nor was there any group-wise numerical trend. Thus, the small correlation between the fNIRS signal and the FDG-PET suggests a potential correspondence between the two that is independent of the diagnostic information contained within the fNIRS signal.

**Table 4:** The LME model result indicates the mean *ΔHbO*_*2*_ from the bilateral DLPFC regions showing a significant positive correlation to the overall FDG-PET.

	fNIRS bilateral DLPFC		
*Predictors*	*Estimates*	*std*. *Beta*	*p-value*
(Intercept)	-0.00	0.01	0.398
Age	-0.00	-0.01	0.950
Global FDG-PET	0.00	0.31	0.007
Random Effects			
σ^2^	0.00		
τ00 group	0.00		
ICC	0.05		
N group	4		
Observations	79		
Marginal R^2^ / Conditional R^2^	0.090 / 0.132		

LME: Linear mixed effect, fNIRS: Functional near-infrared spectroscopy, DLPFC: Dorsolateral prefrontal cortex, FDG-PET: Fluorodeoxyglucose-positron emission tomography, ICC: Intraclass correlation coefficient.

### fNIRS and amyloid PET

When the identical model was fitted to the amyloid PET data, no significant relationship between the fNIRS signal and amyloid deposition was found. The null result was replicated when the simple Pearson correlation between the amyloid PET and fNIRS signals was calculated. Thus, the results suggest that the functional signal acquired from the fNIRS does not capture the amount of amyloid deposition with or without controlling for the subject’s age and group differences in fNIRS activation levels.

**Fig 6:**
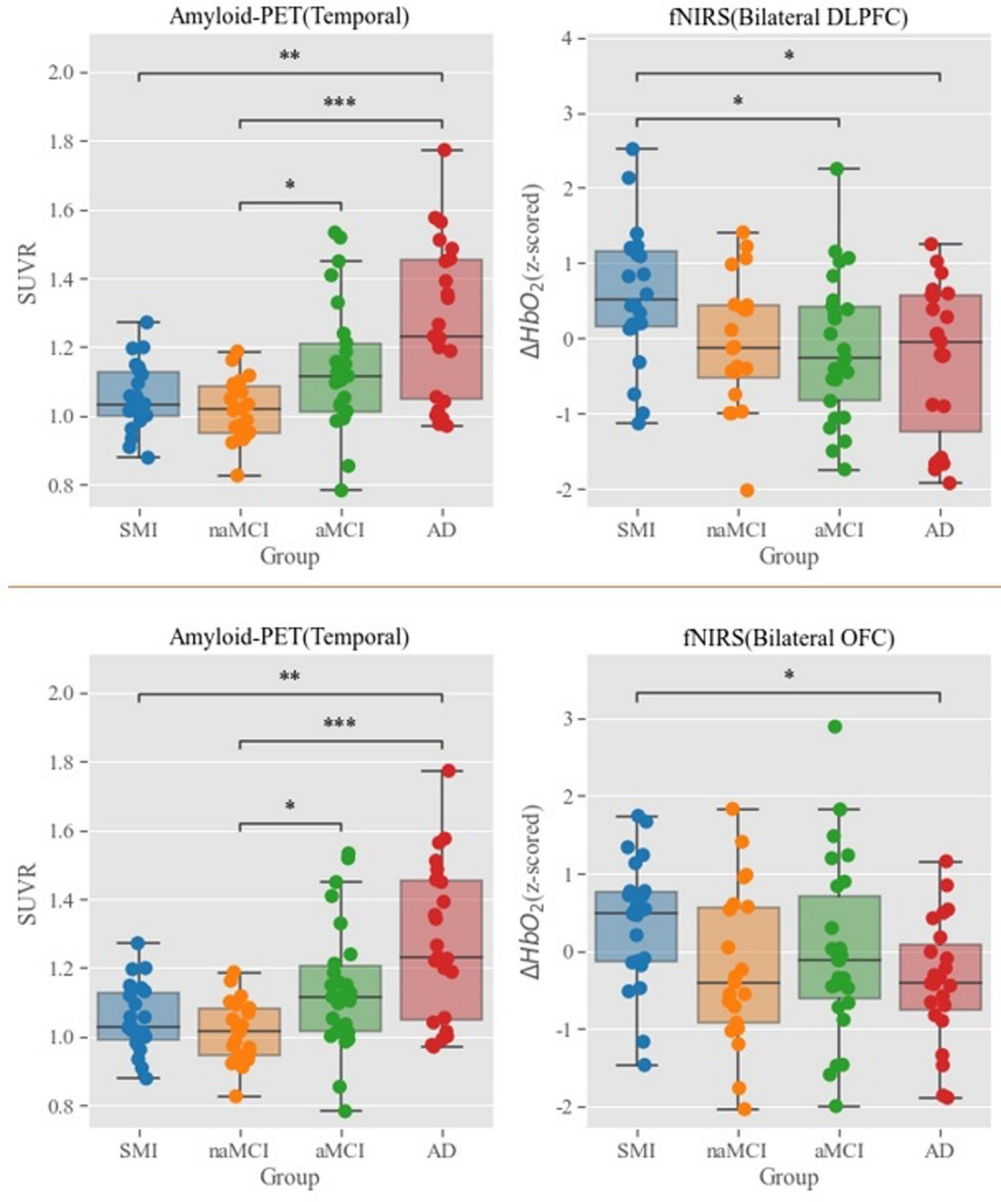
The group effect on Amyloid-PET in the temporal lobe and the mean *ΔHbO*_*2*_ from the two fNIRS ROIs. PET: Positron emission tomography, fNIRS: Functional near-infrared spectroscopy, ROI: Region of interest.

Next, a MANCOVA and subsequent ANOVAs were conducted to investigate the group diagnostic effect on the signals from the two fNIRS ROIs and the amyloid PET signal in the temporal lobe, where the group difference was the most obvious. The MANCOVA across amyloid PET from the temporal lobe and the bilateral DLPFC fNIRS ROIs revealed that the group effect was significant in the MANCOVA [*F*(6, 168) = 6.63, *p* < .001], and the subsequent ANOVAs were significant [amyloid PET: *F*(3, 85) = 10.56, *p* < .001; DLPFC fNIRS: *F*(3, 85) = 3.59, *p* < .017]. Furthermore, the MANCOVA on the same aPET ROI and the bilateral OFC fNIRS revealed a significant group effect [*F*(6, 174) = 6.64, *p* < .001], leading to a significant effect on amyloid PET [*F*(3, 88) = 11.46, *p* < .001] and a marginally significant effect on fNIRS [*F*(3, 88) = 2.63, *p* = .055], Overall, Fig 6 shows that the amyloid PET signal tends to better differentiate the AD group from others, and the fNIRS signal tends to differentiate the SMI group from the other groups.

### fNIRS and anatomical measures

**Fig 7:**
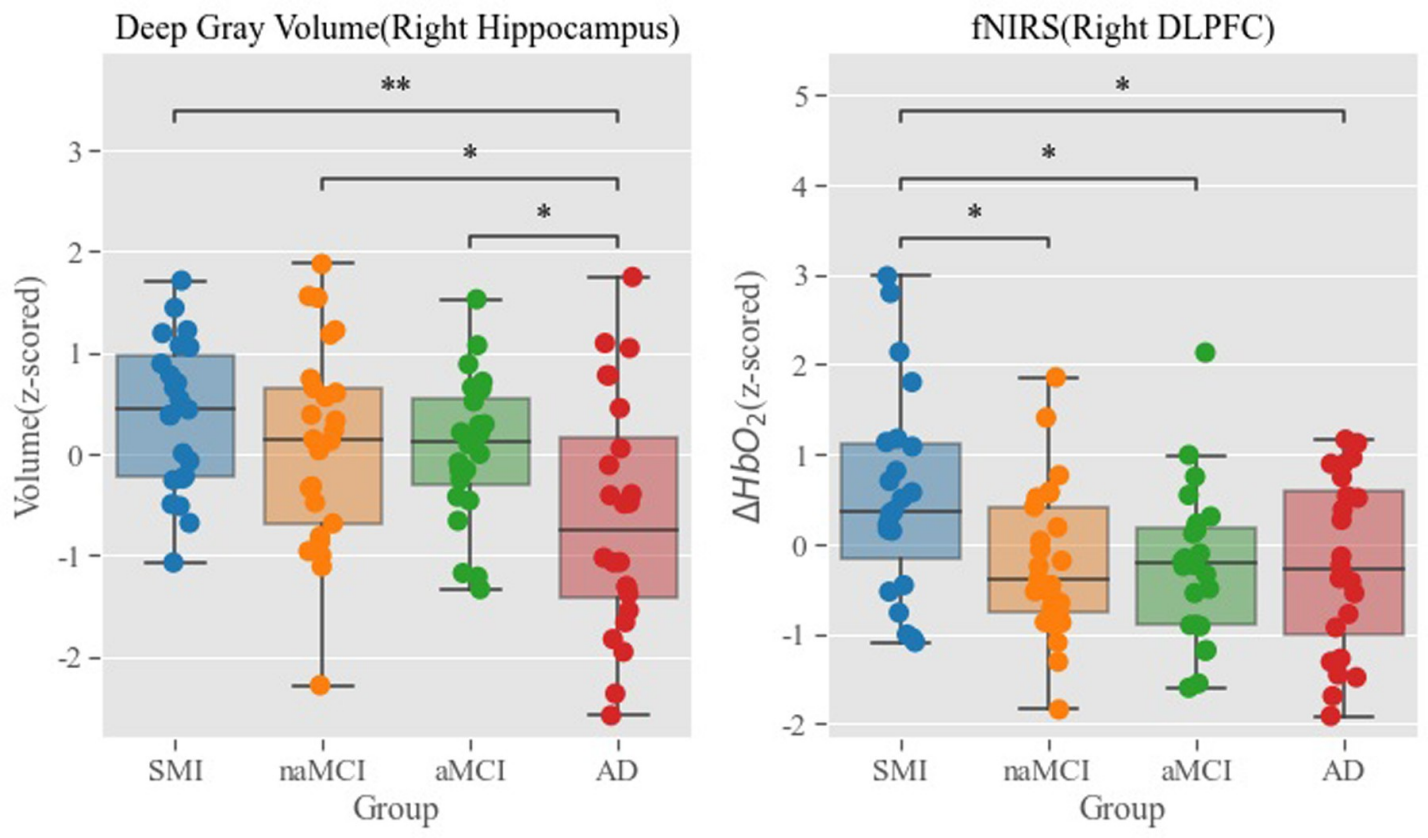
The MANCOVA on the right hippocampal gray volume and right DLPFC fNIRS. MANOVA: Multivariate analysis of covariance, DLPFC: Dorsolateral prefrontal cortex, fNIRS: Functional near-infrared spectroscopy.

When the LME model was fitted to the anatomical measures, no significant correlation between the fNIRS ROIs and gray volume or cortical thickness was found above and beyond what the group difference could explain. Thus, we moved on to the MANCOVA to see whether the group diagnostic effect differed across anatomical changes and fNIRS, where several interesting significant group effects were found for hippocampal gray volume and DLPFC fNIRS ROIs. Specifically, the MANCOVA on the right hippocampal gray volume and right DLPFC fNIRS ROI were significant [*F*(6, 180) = 4.27, *p* < .001], leading to a significant effect on the hippocampal gray volume [*F*(3, 91) = 5.01, *p* < .01] and a marginally significant effect on fNIRS [*F*(3, 91) = 3.48, *p* = .019] (Fig 7). The comparison between the group pairs in each ANOVA revealed a mirrored pattern between the hippocampal volume and hemodynamic response in the DLPFC. That is, similar to what was shown in the amyloid PET results, the hippocampal volume better differentiates the AD subjects from other groups whereas the fNIRS did so for the SMI group compared to the others. This pattern was consistent across hippocampal and DLPFC regions, but the pairwise group difference varied slightly, suggesting that groups underwent differential functional and anatomical changes.

#### fNIRS and frontal cortical thickness

**Fig 8:**
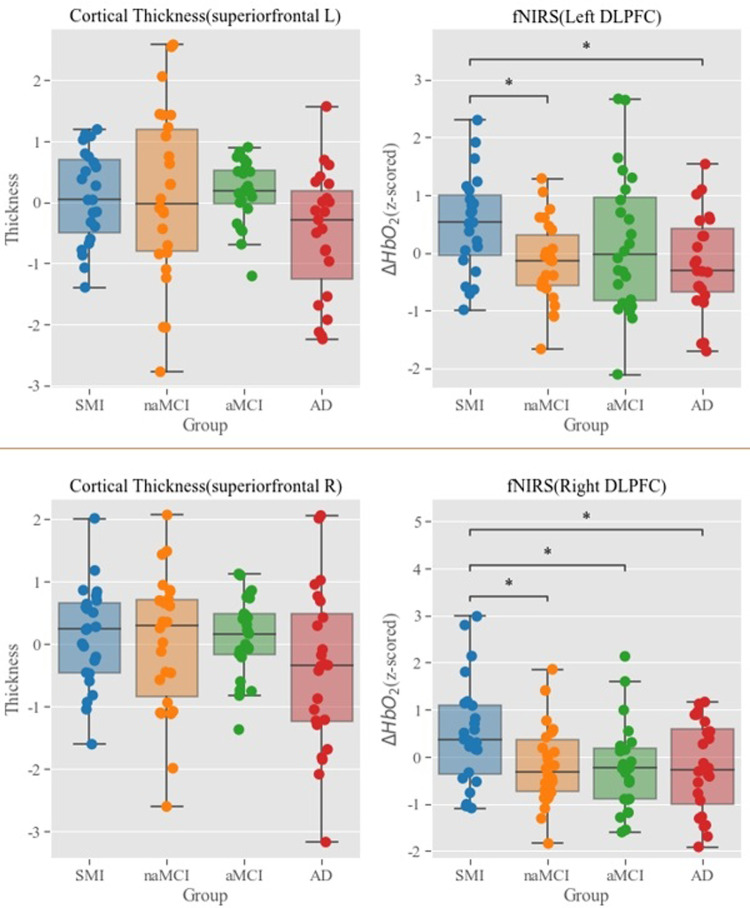
MANCOVA test on the frontal cortical thickness and fNIRS. LME: Linear mixed effect (LME), MANOVA: Multivariate analysis of covariance.

As a supplementary check-up, we also administered the same LME and the MANCOVA test on the thickness data of the same frontal regions that the fNIRS was mainly targeting. No significant correlations were found from the LME model, and MANOVA results show that the hemodynamic difference across groups found from the bilateral DLPFC was not based on the anatomical changes in the same regions, demonstrating the divergence between the functional and anatomical measures as a function of the group diagnosis (Fig 8).

## Discussion

The key finding of our study is that first, the inter-group differences in the hemodynamic response during SVFT showed decreased cortical activation in the AD and MCI groups, compared to the SMI group which is consistent with the results of previous studies [21,31,32]. Thus, the validity of fNIRS in evaluating the hemodynamic responses during a task was demonstrated in the AD and MCI groups in this study. Also, the hemodynamic response of *ΔHbO*_*2*_ from the bilateral DLPFC regions showed a significant positive correlation to the overall FDG-PET. Thus, overall, this result suggests that the metabolic activity accompanying cerebral blood perfusion measured via FDG-PET is weakly but consistently related to the functional hemodynamics measured via fNIRS. Otherwise, no significant correlation between the fNIRS ROIs and gray volume or cortical thickness fNIRS was identified as hemodynamic responses of the amyloid-PET data and anatomical indicators on brain MRI. Based on the results of this study, it can be predicted that fNIRS will provide useful data for early identification of the metabolic changes that distinguish between normal participants and MCI or AD patients. However, regarding the progression of AD, it is considered at a stage where no conclusions can be made about the usefulness of amyloid imaging with PET and measuring cortical thickness, which has been reported as suitable biomarkers, and functional hemodynamic data through fNIRS [33].

A few previous studies have reported that the correlation between neural activity and blood oxygenation level-dependent responses measured with fNIRS and functional MRI (fMRI) is positive [34–36]. Given that fNIRS only assesses the cortex, it is necessary to determine whether the hemodynamic changes it detects and the metabolic changes and anatomical measures have a correlation. In addition, amyloid PET detects amyloidosis in vivo, and it has a high negative predictive value for AD [37]. FDG-PET allows the detection of topographical information about neurodegeneration by analyzing patterns of hypometabolism and is useful for disease staging and prediction of disease progression [38]. It is believed that confirming its relationship with high task-related hemodynamic activation detected by fNIRS is important in these patients.

A previous study involving healthy participants that investigated the relationship between brain metabolism assessed using FDG-PET and hemodynamic response (which is related to brain activity), assessed using fMRI, showed that the correlations between the regional cerebral metabolic rate of glucose and fMRI metrics were significant [39]. Thus, it is believed that it is important to examine the correlation between hemodynamic response in a particular brain region and the metabolic status of the brain. In our study, the metabolic activity measured via FDG-PET was weak but consistently related to the functional hemodynamics measured via fNIRS. Otherwise, the group diagnostic effect on the global FDG-PET did not differ across groups, nor was there any group-wise numerical trend. Thus, the small correlation between the fNIRS signal and FDG-PET suggests a potential correspondence between the two that is independent of the diagnostic information contained within the fNIRS signal. In addition, the overall amyloid PET signal tends to better differentiate the AD group from other groups, and the fNIRS signal tends to differentiate the SMI group from other groups; this has clinical significance because the potential of aMCI advancing to AD could be determined indirectly based on PET findings in the brain regions.

Gray matter atrophy is a cardinal sign of neurodegeneration [40]. In a previous study, there was a correlation between hippocampal volume and the severity of cognitive impairment, suggesting that the measurement of the hippocampal volume may predict disease progression [41]. In this study, the comparison between the group pairs revealed a mirrored pattern between the hippocampal volume and hemodynamic response in the DLPFC. It is similar to the amyloid PET results, which showed that the hippocampal volume better differentiated the AD group from other groups, whereas the fNIRS differentiated the SMI group from other groups. This pattern was consistent across the hippocampal and DLPFC regions, with only a slight variation in the pairwise group difference, suggesting that the groups underwent differential functional and anatomical changes. The anatomical changes occur only after the participants are diagnosed with AD; however, for the same participants, the functional changes observed via fNIRS occur way earlier as they reveal the cognitive impairment (MCI ∼ AD), implying that fNIRS can be a useful tool for detecting the early risk of cognitive impairment, leading to the preventive medication prescription mentioned in the introduction section.

A limitation of our study is that fNIRS and PET were not performed simultaneously. However, it is thought that the interval between the tests was not long enough for clinical severity to change. Another limitation was that we did not vary the task during fNIRS. SVFT is a tool that is easy to use. It effectively assesses frontotemporal and frontal cortex activation [42], and it is one of the representative tasks used during fNIRS. Thus, we believe it was an appropriate choice for this study.

## Conclusions

The hemodynamic response detected by fNIRS showed a significant correlation with the metabolic changes observed on PET. Therefore, fNIRS may be useful as a screening tool to predict the hemodynamic and metabolic statuses of the brain in patients with MCI and AD.

## Supporting information

S1 Dataset (XLSX)
